# Intramuscular Myxoma in the Supinator Muscle with Transient Postoperative Posterior Interosseous Nerve Palsy: A Case Report

**DOI:** 10.2174/1874325001812010353

**Published:** 2018-08-31

**Authors:** Toshihiro Nonaka, Motoki Sonohata, Shuhei Takeshita, Yosuke Oba, Yoshimasa Fujii, Masaaki Mawatari

**Affiliations:** Department of Orthopaedic Surgery, Faculty of Medicine, Saga University, 5-1-1 Nabeshima, Saga 849-8501, Japan

**Keywords:** Intramuscular myxoma, Supinator muscle, Posterior interosseous nerve

## Abstract

**Background:**

Intramuscular myxomas are rare, benign mesenchymal tumors in the musculoskeletal system, and usually, the tumors arise in the large muscles of the thigh, buttocks, shoulder, and upper arm. However, a tumor of the forearm is very rare. Herein, we describe the case of an intramuscular myxoma in the supinator muscle of a 56-year-old female patient.

**Case Presentation:**

Magnetic resonance imaging showed a well-defined mass that was hypointense with the peritumoral fat ring sign. The differential diagnoses might have been myxoma, schwannoma, or intramuscular hemangioma. The histopathological image showed abundant myxoid tissue, hypocellularity, and poor vascularization. The cells of the tumor were spindle and stellate-shaped with normochromic nuclei. Based on these findings, the pathological diagnosis was an intramuscular myxoma. After excising the tumor, the patient had transient posterior interosseous nerve palsy.

**Conclusion:**

This tumor is curative by resection in toto; however, when the tumor exists in the forearm, surgeons should be careful to avoid damaging surrounding tissues because the tumor is very hard and relatively large compared to the forearm.

## 1 INTRODUCTION

Intramuscular myxomas are rare, benign mesenchymal tumors in the musculoskeletal system. They have an incidence of 0.1 to 0.13 per 100 000 persons [1] and occur more frequently in women; additionally, most patients present with intramuscular myxomas between the fourth and seventh decades of life [2]. Intramuscular myxomas arise in the large muscles of the thigh, buttocks, shoulder, and upper arm [3]. However, a tumor of the forearm is very rare [4]. We describe a rare case of an intramuscular myxoma in the supinator muscle with transient posterior interosseous nerve palsy. We obtained informed consent from this patient to publish her case.

## 2 CASE PRESENTATION

A 56-year-old female patient was referred to our hospital for a mass lesion in her forearm that had been growing slowly, and she had no history of trauma, infection, fever, or weight loss. During medical history taking, we found that a needle biopsy was performed 4 years ago in another hospital, and the result of the cytology examination was class II. The patient also complained of extension lag of the metacarpophalangeal joint of the middle finger that had gradually proceeded without sensory loss. The physical examination revealed an ill-defined area of swelling at the middle part of the forearm. The palpable mass was swollen, measured about 30×30 mm, was smooth and fixed without tenderness or the Tinel’s sign, and had very hard elasticity.

**Fig. (1):**
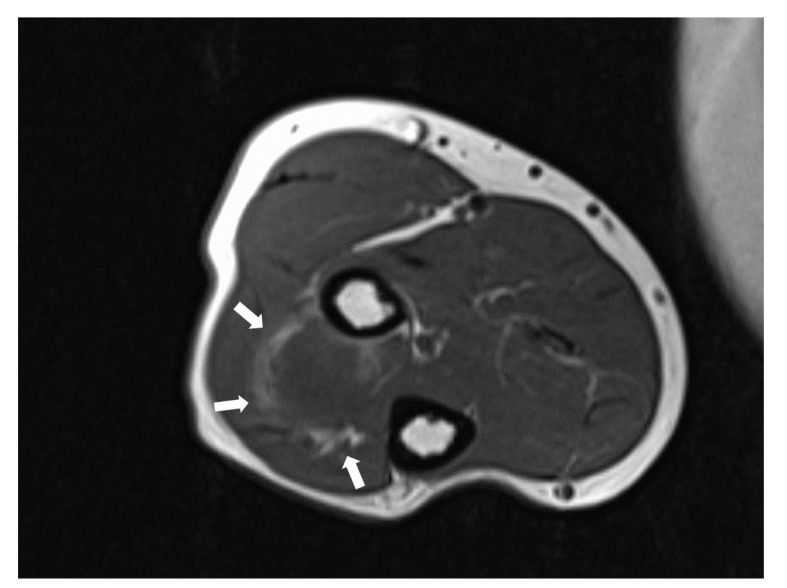
Horizontal slice of the T1-weighted image showing the hypointense signal change of the right supinator muscle. White arrows show the peritumoral fat ring sign.

**Fig. (2):**
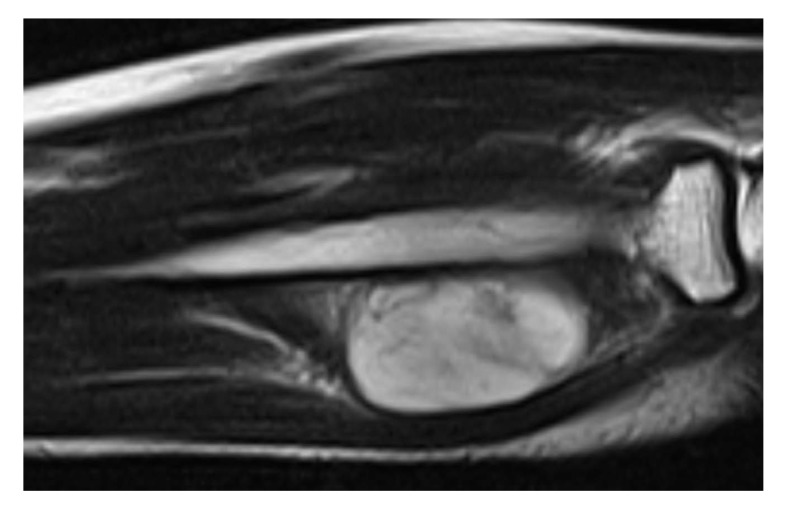
Sagittal slice of the T2-weighted image showing the hyperintense signal change of the right supinator muscle.

**Fig. (3):**
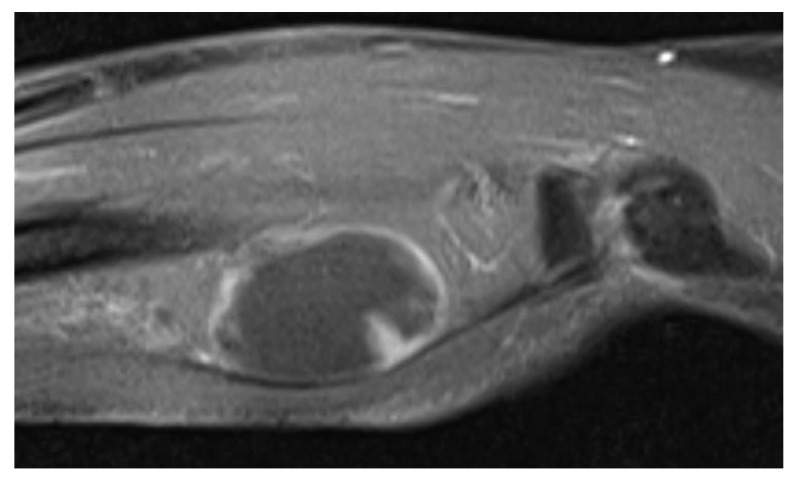
Sagittal slice of the gadolinium-enhanced T1-weighted image showing heterogeneous enhancement of the right supinator muscle and revealing peripheral enhancement of the mass with linear stranding inside the tumor.

The patient was evaluated with radiography, Computed Tomography (CT), and Magnetic Resonance Imaging (MRI). On the x-ray, there was no scalloping, osteolysis, pathological fracture of the radius or ulna, or calcification of soft tissue. On the CT scan, the mass had a low density within the right supinator muscle. The MRI scan of the right forearm revealed a 37×22×27-mm well-defined mass that was hypointense; additionally, the peritumoral fat ring sign was indicated on T1-Weighted Images (T1WI) (Fig. **1**), and a hyperintense area was shown on T2-weighted images (T2WI) (Fig. **2**). Intravenous Gadopentetate Dimeglumide of Gadolinium (GDG) enhancement revealed peripheral enhancement of the mass with linear stranding inside the tumor (Fig. **3**). The imaging findings characterized the mass as intermuscular myxoma; the differential diagnosis might have been myxoma, schwannoma, or intramuscular hemangioma.

Surgical resection was performed under general anesthesia. Local surgical excision of the tumor was performed over the tumor site between the musculus extensor carpi radialis brevis and extensor digitorum communis, taking care to avoid damaging the posterior interosseous nerve. The mass was a well-capsulated cystic, solid, well-defined mass with hard elasticity in the supinator muscle, and it was excised in an en bloc manner from the normal muscle.

**Fig. (4):**
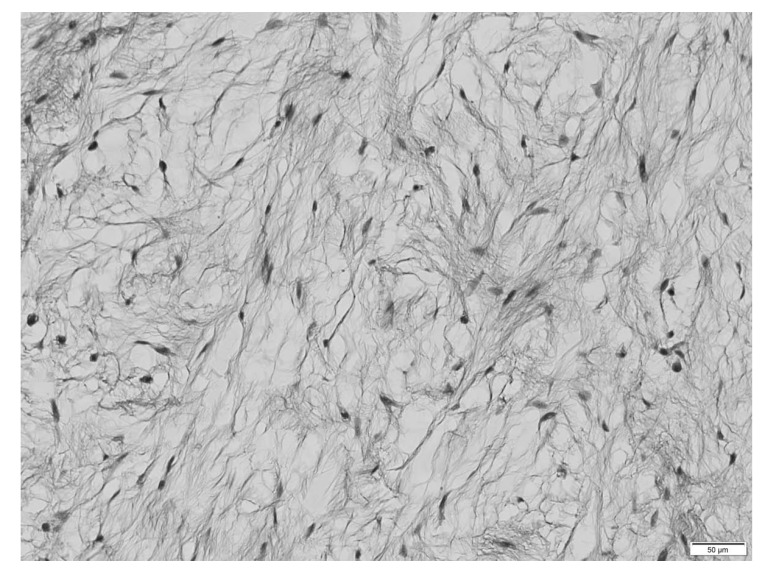
Histopathological image showing abundant myxoid tissue, hypocellularity, and poor vascularization. The cells of the tumor are spindle or stellate shaped with normochromic nuclei (hematoxylin-eosin staining, ×100).

During the histological examination, a cut section of the mass showed glossy grey-white glistening gelatinous areas macroscopically. Microscopy showed that the cells were spindle and stellate-shaped, and collagen fiber was in abundance in the mucoid hypocellular material of neoplasms with low vascularity (Fig. **4**). Mitotic activity, necrosis, and nuclear atipias were not seen. Based on these findings, the pathological diagnosis was an intramuscular myxoma.

Postoperatively, there was no problem during the process of wound healing. However, she had difficulty extending the metacarpophalangeal joint of the right fingers without sensory disturbance. The Manual Muscle Testing System [5] showed that the muscle power values of the extensor digitorum, extensor digiti minimi, extensor indicis, extensor pollicis longus, and extensor carpi ulnaris were 0. The muscle power value of the extensor carpi radialis was 3.

She did not undergo an electrophysiological examination; however, on the basis of findings from physical examinations, we made a diagnosis of posterior interosseous nerve palsy due to compression during the operation. We prescribed an orthosis for functional recovery of her fingers. Three months postoperatively, the posterior interosseous nerve palsy improved completely. She has not shown any signs of recurrence detected by MRI 1 year later.

## 3 DISCUSSION

Myxomas are benign connective tissue tumors from stellate mesenchymal cells that lose their capacity to produce collagen, and they occur at various locations, such as the heart and bones [2]. In 1948, Stout described the diagnostic criteria of myxomas initially [2]. Intramuscular myxomas arise from skeletal muscles, and they were described as a distinct subtype of myxomas [6]. Intramuscular myxomas usually occur between the fourth and seventh decades of life and more frequently in women [7]. Intramuscular myxomas usually manifest as a single mass; however, several intramuscular myxomas have manifested with fibrous dysplasia as part of Mazabraud’s syndrome [8]. Intramuscular myxomas commonly occur in the thighs, buttocks, shoulder muscles, or upper extremities [9]. In contrast, intramuscular myxomas in the forearm region are extremely rare. Following a literature review, it was revealed that only six cases of an intramuscular myxoma in the forearm region and only one case of an intramuscular myxoma in the supinator muscle have been reported previously [4].

Intramuscular myxomas usually have a cystic-like appearance on a radiograph, homogeneous low-density lesion appearance on a CT scan, hypointense appearance on T1WI, and hyperintense appearance on T2WI. Fat or edema in the surrounding muscles, the so-called peritumoral fat ring sign, on T1WI and increased signal in the adjacent muscle on T2WI are diagnostic signs for distinguishing intramuscular myxomas from other myxoid tumors. Additionally, GDG may show peripheral and internal enhancement [10]. In the current case, almost all the aforementioned findings were present. Therefore, the diagnosis by imaging was consistent with that of previous reports. In 65%–89% of myxomas, a thin rim of fat (or approaching adipose tissue signal intensity because of volume averaging) is noted most prominently at the superior and inferior poles of the lesion, representing atrophy of the adjacent muscle [11]. The target sign is commonly seen in neurofibroma (58%) but can also be detected in schwannoma (15%). This sign refers to a low to intermediate T2-weighted signal intensity located centrally secondary to fibrous tissue with a higher collagen content and high T2-weighted signal intensity located peripherally, probably related to myxoid (high water content) tissue [12]. If the peripheral myxoid component is predominant, the lesion may be confused with other myxoid lesions, particularly on images obtained with fluid-sensitive sequences. Hence, it is important to distinguish the fat ring sign of myxoma from the target sign of neuroma.

Intramuscular myxomas are usually treated with local surgical excision, and recurrences are very rare. Surgeons have to remove the tumors with a clear margin because local recurrences result from incomplete resection. Our patient had no symptoms of recurrence clinically and radiographically 1 year after resection.

There is only one report of a myxoma in the supinator muscle; the patient had posterior interosseous nerve palsy before surgical resection [4]. In our case, the patient had extension lag of the metacarpophalangeal joint of the middle finger preoperatively, and this symptom might have been due to direct compression on the extensor muscle by the tumor or the posterior interosseous nerve palsy. The posterior interosseous nerve palsy postoperatively was caused by excessive retracted soft tissue around the tumor during the operation because the tumor was very hard and relatively large compared to the forearm. If an intramuscular myxoma exists in small muscles, such as the forearm, surgeons should pay maximum attention to avoid damaging the surrounding soft tissues. Therefore, accurately identifying the location and size of the intramuscular myxoma preoperatively is important.

## CONCLUSION

We described the rare case of an intramuscular myxoma in the supinator muscle. The tumor is curative by resection in toto; however, when the tumor exists in the forearm, surgeons should be careful to avoid damaging surrounding tissues.
